# Association of Right Ventricular Pressure and Volume Overload with Non-Ischemic Septal Fibrosis on Cardiac Magnetic Resonance

**DOI:** 10.1371/journal.pone.0147349

**Published:** 2016-01-22

**Authors:** Jiwon Kim, Chaitanya B. Medicherla, Claudia L. Ma, Attila Feher, Nina Kukar, Alexi Geevarghese, Parag Goyal, Evelyn Horn, Richard B. Devereux, Jonathan W. Weinsaft

**Affiliations:** 1 Greenberg Cardiology Division/Department of Medicine, Weill Cornell Medical College, New York, New York, United States of America; 2 Department of Radiology, Weill Cornell Medical College, New York, New York, United States of America; 3 Memorial Sloan Kettering Cancer Center Department of Medicine, New York, New York, United States of America; University of Minnesota, UNITED STATES

## Abstract

**Background:**

Non-ischemic fibrosis (NIF) on cardiac magnetic resonance (CMR) has been linked to poor prognosis, but its association with adverse right ventricular (RV) remodeling is unknown. This study examined a broad cohort of patients with RV dysfunction, so as to identify relationships between NIF and RV remodeling indices, including RV pressure load, volume and wall stress.

**Methods and Results:**

The population comprised patients with RV dysfunction (EF<50%) undergoing CMR and transthoracic echo within a 14 day (5±3) interval. Cardiac structure, function, and NIF were assessed on CMR. Pulmonary artery systolic pressure (PASP) was measured on echo. 118 patients with RV dysfunction were studied, among whom 47% had NIF. Patients with NIF had lower RVEF (34±10 vs. 39±9%; p = 0.01) but similar LVEF (40±21 vs. 39±18%; p = 0.7) and LV volumes (p = NS). RV wall stress was higher with NIF (17±7 vs. 12±6 kPa; p<0.001) corresponding to increased RV end-systolic volume (143±79 vs. 110±36 ml; p = 0.006), myocardial mass (60±21 vs. 53±17 gm; p = 0.04), and PASP (52±18 vs. 41±18 mmHg; p = 0.001). NIF was associated with increased wall stress among subgroups with isolated RV (p = 0.005) and both RV and LV dysfunction (p = 0.003). In multivariable analysis, NIF was independently associated with RV volume (OR = 1.17 per 10 ml, [CI 1.04–1.32]; p = 0.01) and PASP (OR = 1.43 per 10 mmHg, [1.14–1.81]; p = 0.002) but not RV mass (OR = 0.91 per 10 gm, [0.69–1.20]; p = 0.5) [model χ^2^ = 21; p<0.001]. NIF prevalence was higher in relation to PA pressure and RV dilation and was > 6-fold more common in the highest, vs. the lowest, common tertile of PASP and RV size (p<0.001).

**Conclusion:**

Among wall stress components, NIF was independently associated with RV chamber dilation and afterload, supporting the concept that NIF is linked to adverse RV chamber remodeling.

## Introduction

Right ventricular (RV) adverse remodeling is an important prognostic marker that has been linked to heart failure and death [1, 2]. Increased RV size, an adaptation to maintain stroke volume in the context of contractile dysfunction, can ultimately impair systolic function due to altered chamber geometry and increased wall stress. Increased afterload can also stimulate RV dilation, impair systolic function and augment wall stress. Adverse RV remodeling can be reversible in some patients and progressive in others—with the latter possibly due to irreversible fibrosis as caused by increased wall stress and/or impaired perfusion. Myocardial fibrosis has been well studied in relation to left ventricular (LV) remodeling [3–5]. However, the relative associations of RV volume and pressure overload with myocardial fibrosis are unknown. Cardiac magnetic resonance (CMR) is a well-validated reference for RV structure and function, as well as myocardial infarction/fibrosis [6, 7]. “Non-ischemic fibrosis” (NIF)–typically appearing within the mid myocardial or epicardial aspect of the interventricular septum—has been associated with heart failure severity and adverse outcomes in a broad array of cohorts [5, 8, 9]. Among patients with LV systolic dysfunction, prior research has shown NIF to be linked to increased LV wall stress [4, 10] and associated with LV dilation independent of heart failure etiology. NIF has also been widely reported in patients with RV dysfunction [8, 11–13], but its association with RV wall stress is unknown. Regarding RV remodeling, whereas prior studies have shown NIF to be associated with increased afterload in those with pulmonary hypertension [12], the additive impact of RV chamber dilation and myocardial hypertrophy on NIF formation has not been thoroughly investigated. This study examined a broad cohort of patients with RV dysfunction, so as to identify association between NIF and RV wall stress, as well as its constitutive parameters of RV pressure load and volume.

## Materials and Methods

The study protocol was approved by the Weill Cornell Institutional Review Board. CMR and echocardiogram were performed for clinical purposes and data was retrospectively anonymized with written inform consent for use of anonymized data waived.

### Population

The study population was comprised of patients with CMR-evidenced RV systolic dysfunction (RVEF <50%) in whom echo was performed within a 14 day (mean 5±3) interval for quantification of PA pressure. Patients with conditions known to preclude assessment of localized late gadolinium enhancement (e.g. amyloid, hypertrophic cardiomyopathy, septal myocardial infarction) or accurate echo quantification of pulmonary arterial pressure (e.g. inadequate tricuspid regurgitation Doppler envelope, RV outflow tract obstruction, prosthetic pulmonary valve) were excluded. No other patients were excluded based on clinical condition or indication for CMR.

Demographic indices were categorized using a standardized patient questionnaire; results were confirmed via review of medical records. RV dysfunction etiologies were assigned blinded to CMR results, including NIF status. This study was conducted with approval of the Weill Cornell Medical College Institutional Review Board.

### Imaging Protocol

#### Cardiac Magnetic Resonance

CMR was performed using 1.5 Tesla scanners (General Electric, Waukesha, WI). Exams consisted of two components: (1) cine-CMR for geometry/function and (2) delayed enhancement (DE-) CMR for tissue characterization. Cine-CMR was performed using a steady-state free precession sequence. DE-CMR was performed 10–30 minutes after administration of gadolinium (0.2 mmol/kg) using a segmented inversion recovery sequence [14], for which inversion time was tailored to null viable myocardium. Cine- and DE-CMR were obtained in matching LV short and long-axis planes. Non-ischemic fibrosis (NIF) was identified via DE-CMR, on which it was defined in accordance with prior research as localized hyperenhancement within the RV insertion points or along the mid-myocardial aspect of the interventricular septum [9, 11, 15]. NIF pattern was further stratified based on isolated RV insertion site, isolated mid-myocardial, or concomitant insertion site and mid-myocardial late gadolinium enhancement (LGE).

Cardiac chamber geometry and function were quantified via cine-CMR. RV end-diastolic and end-systolic chamber volumes were measured in contiguous short axis images using a validated automated segmentation algorithm [16], with results used to calculate RV ejection fraction (EF). RV mass and volume (mass * specific gravity) were quantified via planimetry of epicardial and endocardial borders. Linear indices of RV chamber size, function, and wall thickness were measured in accordance with established methods used in prior population-based studies [17] Tricuspid regurgitation was graded based on jet size in relation to the right atrium (moderate ≥ RA area).

#### Echocardiography

Transthoracic 2D echocardiograms were acquired using commercially available equipment (General Electric Vivid-7 [Milwaukee, WI], Siemens SC2000 [Malvern, PA], Philips ie33 [Andover, MA]). Pulmonary artery systolic pressure (PASP) was calculated using the modified Bernoulli equation (4 [peak tricuspid regurgitant velocity]^2^ + right atrial pressure), for which tricuspid regurgitant peak velocity was measured via Doppler and right atrial pressure determined based on size and collapsibility of the inferior vena cava [18].

#### Wall Stress

RV wall stress (σ**)** was calculated using an established formula [19] that incorporates RV end-systolic chamber volume (RVESV), PASP, and RV myocardial volume.

$$\text{RV}\;\text{wall}\;\text{stress}\;=\;\frac{\text{PASP}\;*\;\text{RVESV}}{\text{RV}\;\text{myocardial}\;\text{volume}}$$

In accordance with prior methods [19], RV systolic pressure was estimated by PA systolic pressure, end-systolic radius calculated based on assumptions of spherical chamber geometry, and RV systolic wall thickness calculated via planimetry of the RV free wall.

### Statistical Methods

Continuous variables (expressed as mean ± standard deviation) were compared using Student’s t-tests for two group comparisons, and analysis of variance (ANOVA) for multiple group comparisons. Categorical variables were compared using Chi-square or, when fewer than 5 expected outcomes per cell, Fisher’s exact test. NIF was assessed in relation to RV afterload and structural parameters using univariable and multivariable logistic regression analyses. Two-sided p <0.05 was considered indicative of statistical significance. Statistical calculations were performed using SPSS 20.0 (SPSS Inc, Chicago, IL).

## Results

### Population characteristics

118 patients with RV systolic dysfunction were studied, among whom 47% had NIF. Among patients with NIF (n = 56), 52% (n = 29) had isolated RV insertion site, 23% (n = 13) had mid-myocardial, and 25% (n = 14) had concomitant RV insertion and mid-myocardial LGE on DE-CMR.

Table 1 details clinical characteristics of the study population. As shown, patients with NIF more commonly had clinically evident RV decompensation, as evidenced by need for inotropic or loop diuretic therapy (both p<0.05). Regarding clinical diagnoses, NIF was more common among patients with idiopathic pulmonary hypertension (p = 0.004) but was also present among those with other RV-associated cardiomyopathies.

**Table 1:** Clinical Characteristics.

Parameter	Overall (n = 118)	NIF + (n = 56)	NIF - (n = 62)	P
Age (year)	55±18	55±18	54±17	0.72
Gender (male)	65% (77)	59% (33)	71% (44)	0.17
Coronary Revascularization	16% (19)	13% (7)	19% (12)	0.31
Myocardial Infarction	10% (12)	5% (3)	15% (9)	0.09
**Congenital Heart Disease**	9% (11)	5% (3)	13% (8)	0.16
Tetralogy of Fallot	4% (5)	2% (1)	7% (4)	0.37
Atrial septal defect	3% (3)	2% (1)	3% (2)	1.0
Parenchymal Lung Disease	8% (9)	5% (3)	10% (6)	1.00
Idiopathic pulmonary hypertension	7% (8)	14% (8)	-	**0.004**
Connective Tissue/Rhematologic Disease	4% (5)	7% (4)	2% (1)	0.19
**Heart Failure Medication Regimen**				
Beta-blocker	59% (70)	55% (31)	63% (39)	0.96
ACE-Inhibitor/Angiotensin Receptor Blocker	39% (46)	39% (22)	39% (24)	0.54
Loop diuretic	47% (56)	59% (33)	37% (23)	**0.002**
Inotropic support*	22% (26)	34% (19)	11% (7)	**0.003**
Pressor support†	19% (22)	14% (8)	23% (14)	0.25
**Hemodynamic Indices**				
Heart Rate (bpm)	84±18	85±16	84±19	0.75
Systolic blood pressure (mmHg)	116±18	115±19	117±18	0.73
Diastolic blood pressure (mmHg)	73±13	73±14	73±13	0.80
**Anthropomorphic indices**				
Height (cm)	172±11	172±11	172±11	0.91
Weight (kg)	81±21	83±19	80±23	0.50
Body surface area (m^2^)	1.9±0.2	1.9±0.2	1.9±0.2	0.52

Boldface type indicates p < 0.05.

* milrinone or dobutamine, within 90 days of CMR

^†^ norepinephrine, epinephrine, vasopressin, phenylephrine, or dopamine within 90 days of CMR

**Fig 1:**
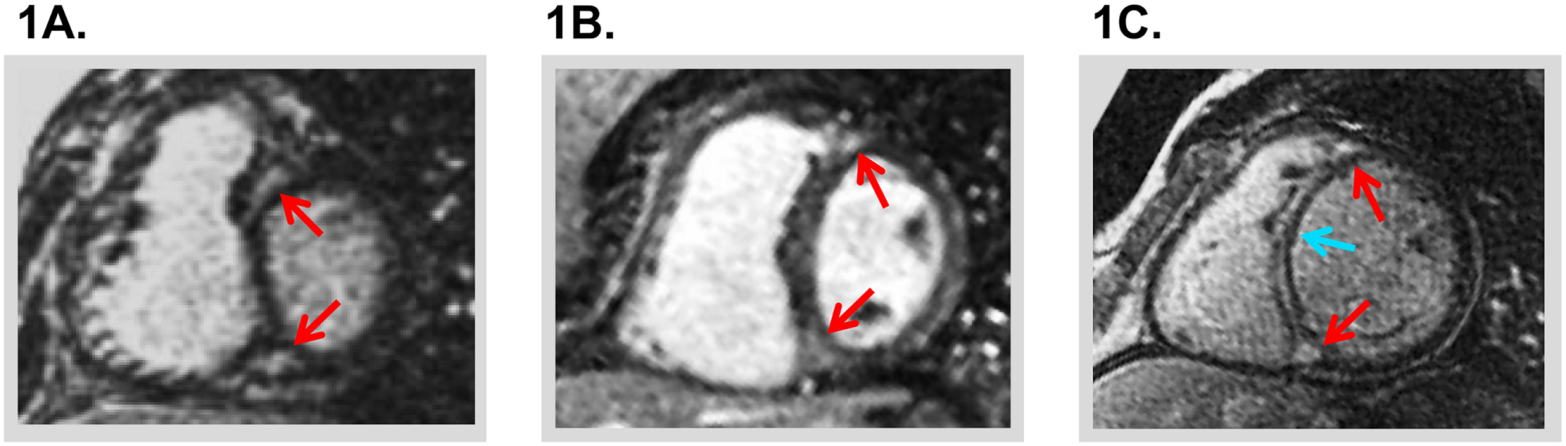
Typical Examples. Representative examples of NIF in patients with adverse right ventricular (RV) remodeling. **1A** and **1B** demonstrate RV insertion site hyperenhancement (red arrows) with 1A demonstrating NIF in context of concomitant RV chamber dilation and myocardial hypertrophy, whereas patient 1B demonstrating NIF with RV chamber dilation alone. **1C** demonstrates NIF involving both the RV insertion site and interventricular septum (blue arrow).

Fig 1 provides representative examples of NIF among study participants with different patterns of RV geometry.

### Cardiac Chamber Remodeling

Table 2 reports cardiac functional and structural indices stratified based on NIF. As shown, all LV parameters were similar between groups. Conversely, NIF was associated with decreased RV ejection fraction (p = 0.01) and increased RV chamber volumes (all p≤0.01).

**Table 2:** Contractile Function / Geometry.

	Overall (n = 118)	NIF + (n = 56)	NIF − (n = 62)	P
***Right Ventricular Function***				
Ejection fraction (%)	37±9	34±10	39±9	**0.01**
Stroke volume (ml)	70±29	72±34	69±24	0.64
(ml/m^2^)	37±15	37±17	37±14	0.84
Cardiac Output (L/min)	5.7±2.0	5.9±2.2	5.5±1.8	0.73
(L-m^2^/min)	3.0±1.0	3.0±1.1	2.9±1.0	0.82
***Right Ventricular Geometry***				
End-systolic volume (ml)	126±62	143±79	110±36	**0.006**
(ml/m^2^)	65±31	74±40	58±19	**0.01**
End-diastolic volume (ml)	196±76	215±95	179±49	**0.01**
(ml/m^2^)	102±39	111±48	95±27	**0.03**
Short axis end-diastolic diameter (cm)	5.4±1.0	5.6±1.2	5.2±0.8	**0.04**
Short axis end-systolic diameter (cm)	4.2±1.0	4.4±1.1	4.0±0.9	**0.03**
Short axis fractional shortening (cm)				
Long-axis end-diastolic diameter (4-chamber; cm)	5.3±1.2	5.6±1.2	5.0±1.1	**0.008**
End-diastolic length (4-chamber; cm)	8.2±1.5	8.2±1.8	8.2±1.2	0.94
Pulmonary annulus diameter (cm)	2.9±0.4	3.0±0.5	2.9±0.3	0.2
Free wall thickness (cm)	0.4±0.1	0.5±0.1	0.4±0.1	**0.01**
Relative wall thickness	0.08±0.03	0.09±0.03	0.08±0.03	0.39
Myocardial mass (gm)	56±19	60±21	53±17	**0.04**
(gm/m^2^)	29±10	31±11	28±9	**0.049**
Relative mass (gm/ml)	0.48±0.13	0.46±0.15	0.50±0.12	0.16
Interventricular septal flattening	50% (59)	68% (38)	34% (21)	**<0.001**
Advanced (≥ moderate) tricuspid regurgitation	35% (41)	48% (27)	23% (14)	**0.003**
***Left Ventricular Function***				
Ejection fraction (%)	40±19	40±21	39±18	0.73
Ejection fraction < 50%	71% (84)	68% (38)	74% (46)	0.45
Stroke volume (ml)	62±23	60±21	65±24	0.27
(ml/m^2^)	32±12	31±10	34±13	0.14
Cardiac Output (L/min)	5.1±1.7	5.0±1.8	5.2±1.7	0.54
(L-m^2^/min)	2.6±0.9	2.5±0.8	2.7±0.9	0.18
***Left Ventricular Geometry***				
End-systolic volume (ml)	125±84	132±102	119±64	0.41
(ml/m^2^)	64±43	66±51	62±34	0.67
End-diastolic volume (ml)	188±84	192±103	183±62	0.59
(ml/m^2^)	97±42	97±50	96±33	0.98
End-diastolic diameter (short axis; cm)	6.1±1.0	6.1±1.2	6.0±0.9	0.64
Septal wall thickness (cm)	1.0±0.3	1.0±0.3	1.0±0.2	0.79
Lateral wall thickness (cm)	0.9±0.2	0.9±0.3	0.9±0.2	0.74
Myocardial mass (gm)	164±60	164±70	165±51	0.94
(gm/m^2^)	85±30	84±34	85±26	0.74

Boldface type indicates p < 0.05

RV linear dimensions similarly demonstrated an association between NIF and RV dilation, as evidenced by increased RV short axis diameter (p = 0.04) and a similar trend for RV long axis (p = 0.08) diameter. Whereas RV mass (p = 0.04) and wall thickness (p = 0.01) were slightly increased among patients with NIF, both RV mass/volume and relative wall thickness (adjusted for short axis diameter) were similar (both p = NS), suggesting absolute increments in RV mass as a response to chamber enlargement.

### RV Pressure Overload

Echo-quantified PASP was higher among patients with NIF (52±18 vs. 41±18 mmHg, p = 0.001), paralleling increased prevalence of pulmonary hypertension (75% vs. 47%, p = 0.002) as defined via an echo-based established cutoff (≥40 mmHg)[20].

Cine-CMR also demonstrated evidence of increased RV afterload in association with NIF. Septal flattening was 2-fold more common among patients with NIF (68% vs. 34%, p<0.001); flattening occurred during both systole and diastole in nearly all affected patients (96%) suggesting concomitant RV pressure and volume overload.

### RV Wall Stress

RV wall stress was nearly 1.4 fold higher among patients with NIF (17±7 vs. 12±6 kPa; p<0.001), consistent with parallel increments in RV chamber volume and afterload. RV wall stress was higher among sub-groups of patients with RV insertion site (16±6 vs. 12±6 kPa p = 0.002), mid-myocardial (16±7 vs. 12±6 kPa, p = 0.03), and concomitant RV insertion site and mid-myocardial NIF (18±9 vs. 12±6 kPa, p = 0.002), compared to those without NIF. Wall stress values were similar between affected patients partitioned based on NIF pattern (p = 0.97).

**Fig 2:**
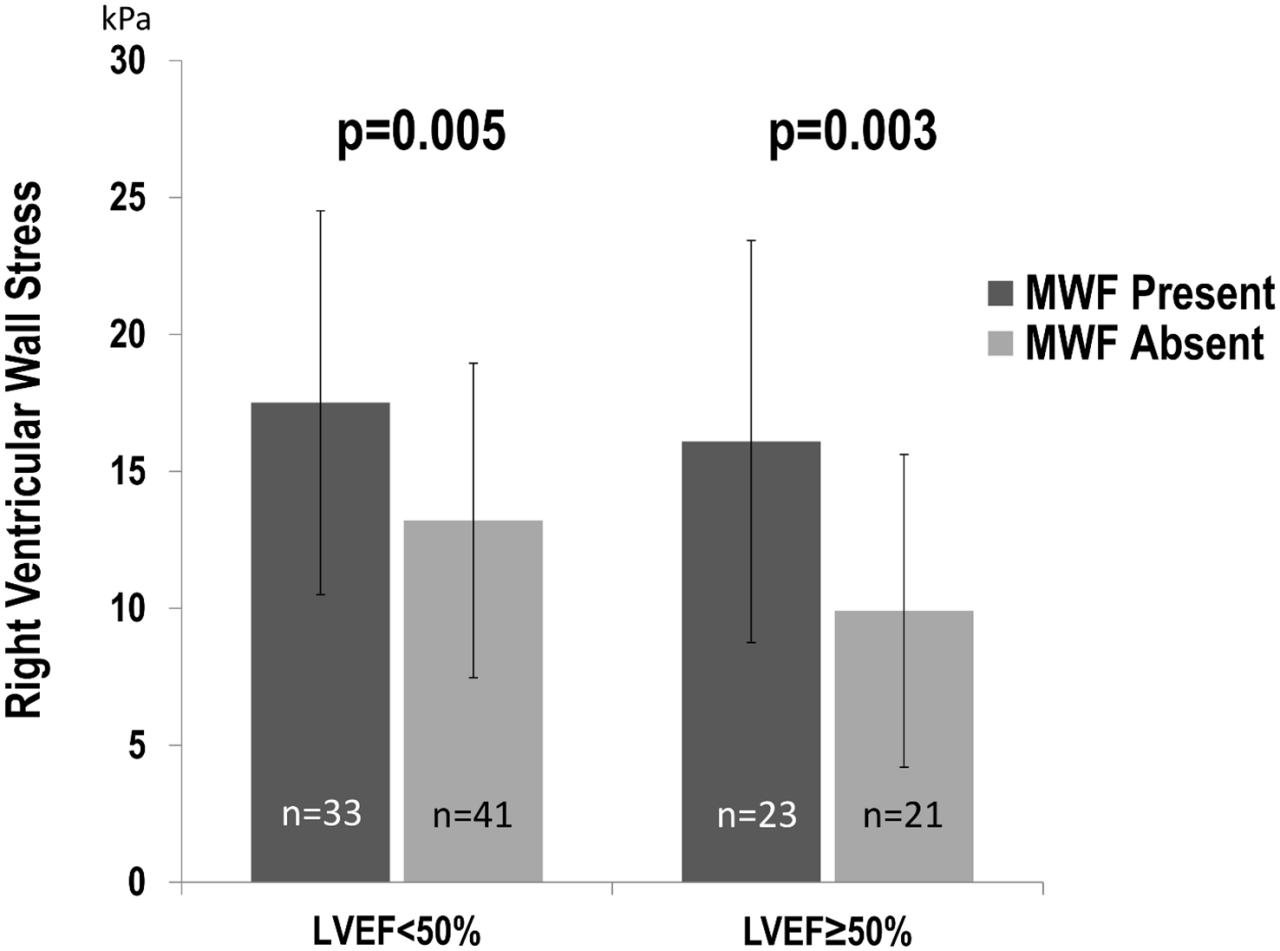
Right Ventricular Wall Stress in Relation to NIF. RV wall stress (mean SD) compared between patients with and without NIF, among study sub-groups with preserved (EF > 50%) and impaired (EF < 50%) LV systolic function. Note that within both strata of LV function, NIF was associated with higher RV wall stress (both p<0.01).

As shown in Fig 2, NIF-associated increments were of greatest magnitude among patients with isolated RV dysfunction (p = 0.005) but also differed significantly among patients with bi-ventricular dysfunction (p = 0.003).

**Fig 3:**
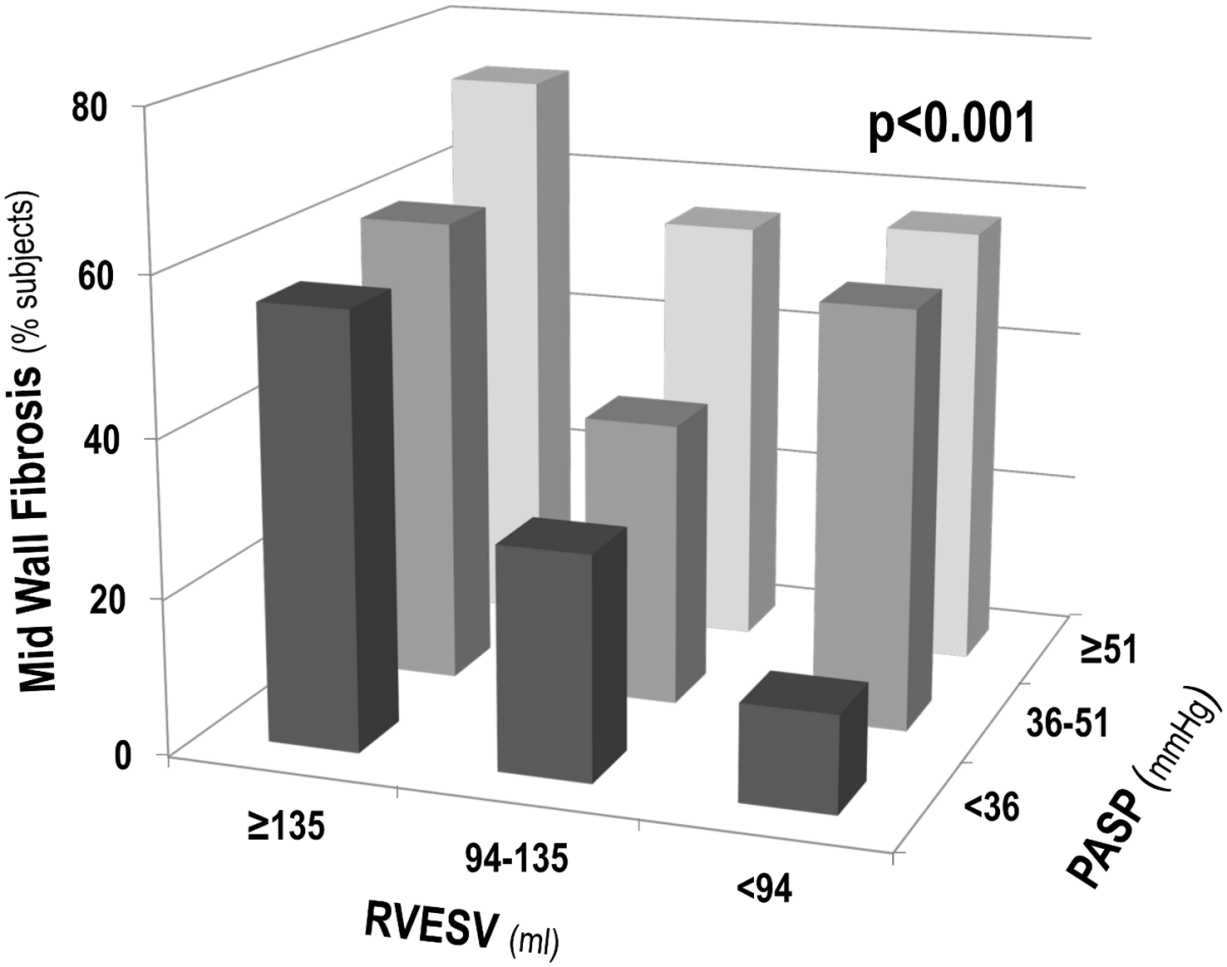
NIF in Relation to RV Wall Stress Components. Prevalence of NIF in relation to population-based tertiles of RV chamber volume and PA pressure. As shown, NIF prevalence was greater among patients within increased strata of each RV remodeling parameter (p<0.001).

Multivariable logistic regression models were constructed to test associations between NIF and individual components of RV wall stress—PASP, RV chamber volume and RV myocardial volume (RV mass). Table 3 reports logistic regression modeling examining NIF in relation to echo-quantified PA pressure as well as CMR quantified RV volumes. As shown, whereas all wall stress components were associated with NIF in univariable analysis, multivariable modeling demonstrated RV volume and PA pressure to be independently associated with NIF even after controlling for RV mass. Of note, the association between NIF and both PA pressure and RV volume was continuous, such that a 20 mmHg increment in PA pressure or a 50 ml increment in RV end-systolic volume would similarly be expected to result in a 2-fold increment in likelihood of NIF. Fig 3 reports prevalence of NIF in relation to population-based tertiles of PA systolic pressure and RV end-systolic volume. Results demonstrate greater prevalence of NIF among patients within increased strata of PA pressure and RV chamber volume (both p<0.001).

**Table 3:** Volumetric Multivariable Regression for Presence of Non-Ischemic Fibrosis.

	Univariable Regression		Multivariable Regression *Model chi-square = 21*.*35*, *p < 0*.*001*	
Variable	Odds Ratio (95% Confidence Interval)	P	Odds Ratio (95% Confidence Interval)	P
PA Systolic Pressure*	1.43 (1.14–1.79)	**0.002**	1.43 (1.14–1.81)	**0.002**
RV End-Systolic Volume*	1.15 (1.05–1.26)	**0.004**	1.17 (1.04–1.32)	**0.01**
RV Myocardial Mass*	1.23 (1.00–1.50)	**0.04**	0.91 (0.69–1.20)	0.50

* per increments of 10 (ml, mg, mmHg respectively)

Table 4 examines corresponding linear components of RV wall stress (chamber diameter, wall thickness) in relation to NIF. As shown, linear RV wall stress components demonstrated comparable relationships with NIF to those of volumetric indices, as evidenced by an independent association between RV end-systolic diameter and NIF (p = 0.01) even after controlling for PASP (p = 0.006) and wall thickness (p = 0.13).

**Table 4:** Linearly-Derived Multivariable Regression for Presence of Non-Ischemic Fibrosis.

	Univariable Regression		Multivariable Regression *Model chi-square = 18*.*10*, *p < 0*.*001*	
Variable	Odds Ratio (95% Confidence Interval)	P	Odds Ratio (95% Confidence Interval)	P
PA Systolic Pressure*	1.43 (1.14–1.79)	**0.002**	1.39 (1.10–1.75)	**0.006**
**RV End-Systolic Diameter** (cm)	1.54 (1.05–2.27)	**0.03**	1.79 (1.14–2.81)	**0.01**
**RV Wall Thickness** (mm)	1.78 (0.48–6.60)	0.39	3.37 (0.71–16.1)	0.13

* per increments of 10 (ml, mg, mmHg respectively)

## Discussion

This study provides several new findings concerning myocardial fibrosis among patients with RV systolic dysfunction. (1) Among a broad population inclusive of differing etiologies of RV systolic dysfunction, NIF was common—identified by CMR in nearly half (47%) of patients. (2) NIF was more common among patients with markers of advanced heart failure, as evidenced by need for diuretic or inotropic therapy (both p<0.01) as well as patients with more advanced RV systolic dysfunction (p<0.001). (3) NIF was strongly associated with RV wall stress, which was higher among NIF patients with isolated RV dysfunction (p = 0.005) as well as those with biventricular failure (p = 0.003). (4) Among wall stress components, RV chamber volume and PA systolic pressure were each independently associated with NIF (p≤0.01), whereas RV mass was not (p = 0.5). NIF prevalence increased stepwise in relation to PA pressure and RV chamber dilation and was > 6-fold more common in the highest, vs. the lowest, common tertile of PASP and RV end-systolic volume (p<0.001).

To the best of our knowledge, this is the first study to examine the independent associations of RV pressure overload and volume on NIF among a diverse population with RV remodeling. Prior CMR studies have shown NIF to be associated with adverse clinical outcomes [8, 21]. However, investigations have been limited to cohorts with particular clinical conditions—such as pulmonary hypertension or congenital heart disease, each of which would be expected to produce specific remodeling stimuli (i.e. primary RV pressure or volume overload) and thereby limit the ability to study NIF across a diverse range of RV geometric patterns. For example, whereas PA pressure was reposted to be independently associated with NIF after controlling for RV volume in a prior study [12], data were derived from a pulmonary hypertension cohort in which increased afterload was the primary determinant of RV remodeling, thereby limiting insight into the role of RV volume alone as a stimulus for NIF. Our results, demonstrating RV pressure overload and volume to be independently associated with NIF, support the notion that NIF results from different remodeling pathways that commonly result in increased wall stress.

Our finding of a strong association between NIF and RV wall stress extends upon prior studies that have examined cardiac chamber remodeling in relation to myocardial tissue properties. Paralleling current data among patients with RV dysfunction, prior research by our group among patients with LV dysfunction has shown NIF to be associated with increased LV wall stress, and independently linked to LV chamber dilation [4]. Concerning RV remodeling, a link between abnormal myocardial substrate and increased RV wall stress has been shown using PET imaging: Among a pulmonary hypertension cohort undergoing FDG-PET and right heart catheterization before and after vasodilator therapy, RV FDG accumulation correlated with wall stress and decreased in proportion to wall stress reduction following treatment with epoprostenol [22]. Regarding mechanism, it is possible that increased wall stress results in impaired balance between myocardial perfusion and energy consumption, resulting in myocardial necrosis in particular “at risk” regions such as the interventricular septum (subject to concomitant systemic pressure as exerted by the adjacent LV). It is also possible that up-regulation of pro-fibrotic signaling pathways contributes to this process. For example, in animal model of hypoxia-induced pulmonary hypertension, expression of atrial natriuretic peptide (ANP)—a vasodilatory peptide secreted in pathologic conditions of increased myocardial load—was found earliest and most prominently in the RV insertion points and the interventricular septum (corresponding to NIF location on CMR)[23]. It is also likely that NIF itself contributes to impaired systolic function and diastolic compliance, resulting in a deleterious cycle that produces further adverse remodeling.

Beyond volumetric analysis, our study assessed linear RV dimensions as a secondary measure of RV remodeling. Linear analysis paralleled volumetric data, as evidenced by an independent association between RV diameter and NIF even after controlling for RW wall thickness and PA pressure. Of course, a key advantage of RV volume concerns the fact that it reflects global, rather than regional, chamber remodeling. It remains uncertain as to the magnitude to which regional variability in RV remodeling impacts wall stress among patients with different conditions. On the one hand, it is possible that in some patients RV regional and global remodeling could change in parallel due to systemic increases in preload or afterload. On the other hand, other patients might demonstrate regional alterations in RV wall stress due to localized RV injury (e.g. coronary ischemia), targeted interventions (e.g. surgical reconstruction), or intrinsic embryologic and geometric differences between different regions (e.g. infundibulum, body) of the RV.

Several limitations should be noted. First, whereas the interval between CMR and echo was short (mean 5±3 days), RV volumes and PA pressure could have varied in the interim between tests and thus influenced study findings. It should also be noted that the majority of our cohort had concomitant RV and LV systolic dysfunction (EF < 50%), such that this is largely a study of NIF among patients with biventricular dysfunction rather than isolated RV failure. Last, echo was used to quantify PA pressure, rather than the reference standard of invasive hemodynamics. On the other hand, echo is well-validated and widely applied for assessment of PA pressure, as evidenced by its prior application for this purpose in several large-scale population-based studies [24, 25]. More broadly, we note that our approach—predicated on use of non-invasive testing—enabled us to study remodeling correlates of NIF among a variety of patients with RV dysfunction, rather than a selected cohort referred for invasive testing.

## Conclusions

The results of this study demonstrate NIF to be independently associated with both RV pressure overload and volume, supporting the concept that NIF is a generalized marker of adverse remodeling stimuli that commonly produce increased RV wall stress.
